# Dr. John B. Hoyer

**Published:** 1914-05

**Authors:** 


**Dr. John B. Hoyer,** Bellevue 1881, died at his home in Middleport, April 3, 1914.

